# The prognostic value and immunological role of angiogenesis-related patterns in colon adenocarcinoma

**DOI:** 10.3389/fonc.2022.1003440

**Published:** 2022-11-11

**Authors:** Weijie Sun, Ying Xu, Baolong Zhao, Min Zhao, Jiaying Chen, Yimin Chu, Haixia Peng

**Affiliations:** ^1^ Digestive Endoscopy Center, Tongren Hospital, Shanghai Jiaotong University School of Medicine, Shanghai, China; ^2^ Qiqihar Medical University, Qiqihar, Heilongjiang, China

**Keywords:** COAD, angiogenesis, prognosis, drug sensitivity, immunotherapy

## Abstract

Colon adenocarcinoma (COAD) is a malignant tumor with a high mortality rate. Angiogenesis plays a key role in the development and progression of cancer. However, in COAD, studies between angiogenesis and prognosis, immune cell infiltration, and personalized treatment guidance are currently lacking. In the present study, we comprehensively assessed 35 angiogenesis-related genes (ARG) and identified key ARGs affecting OS in COAD patients. The ARG Prognostic Index (ARGPI) was constructed based on a univariate Cox regression model and its prognostic value was evaluated in TCGA-COAD, GSE39582, GSE161158 and TRSJTUSM Cohort. We constructed ARGPI as an independent risk factor for OS in COAD patients and combined with clinical parameters to further construct an ARGPI-based nomogram, which showed a strong ability to predict overall survival in COAD patients. High ARGPI is associated with cancer-related and immune-related biological processes and signaling pathways; high TP53 mutation rate; high infiltration of MSC, pericytes, and stromal cells; and more CMS4 subtype. And low ARGPI benefited more from immune checkpoint inhibitor treatment. In addition, we also predicted the sensitivity of different ARGPI groups to common chemotherapeutic and targeted agents. In conclusion, this study constructed an ARGPI based on ARG, which robustly predicted the OS of COAD patients and provided a possible personalized treatment regime for COAD patients.

## Introduction

According to the latest global cancer statistics, Colon adenocarcinoma (COAD) ranks among the highest in incidence and mortality (1). Patients with advanced colorectal cancer (CRC) have a very poor prognosis compared with those with early-stage colorectal cancer (2). At present, the main methods for the treatment of COAD are surgery, radiotherapy and chemotherapy, targeted therapy and immunotherapy. Although advances in understanding of the pathophysiology of COAD have led to increasingly individualized treatment regimens and have resulted in significantly improved overall survival in advanced stage patients, this is far from sufficient relative to early-stage patients (3). Since current treatments for COAD still have many limitations, it is imperative to develop biomarkers that can effectively predict disease progression and individualized treatment response.

Angiogenesis is one of the key conditions for a variety of physiological and pathological activities (4). Pathological angiogenesis in pathological diseases, including cancer, often results in abnormal vascular structure and function, which is one of the important factors in maintaining rapid growth of cancer (4). Because the oxygen, nutrients and growth factors needed to support the growth of cancer cells are all delivered by blood vessels. It is therefore not difficult to understand that tumor cells secrete a large number of pro-angiogenic factors to maintain the formation of vascular networks, albeit disorganized (5). Therefore, inhibition of angiogenesis is a good therapeutic option for the treatment of solid tumors (6, 7). However, current studies mostly target a certain gene or signaling pathway, such as vascular endothelial growth factor (VEGF), fibroblast growth factor and Notch signaling pathway, etc. (8). Unfortunately, anti-angiogenesis cannot completely eliminate tumor cells, which may also be one of the important reasons for cancer drug resistance (9). Therefore, the future will focus on the combination therapy of anti-angiogenic agents with chemotherapy, targeted therapy and immunotherapy (9–11). Current studies have focused on the value of angiogenesis-related genes in the prognosis and immunotherapy of gastric cancer (12). However, no studies have focused on the role of angiogenesis-related genes (ARG) in COAD, including the prognostic value and choice of combination therapy, especially immunotherapy.

Our study is the first to systematically explore the differential expression and prognostic value of angiogenesis-related genes in COAD and further explore the relationship with the immune microenvironment and the impact on the choice of chemotherapy, targeted therapy and immunotherapy. The 3-gene ARG Prognostic Index (ARGPI) was first identified by COAD. Next, we assessed the clinical value of ARGPI and its impact on the immune microenvironment, and finally assessed the clinical value of ARGPI for the effect of immunotherapy. We believe that this study has certain prognostic value and clinical treatment guiding significance for COAD patients.

## Materials and methods

### Patients and tissue samples

From March 2016 to December 2017, 77 surgical specimens after CRC resection were collected at Tongren Hospital, Shanghai Jiao Tong University School of Medicine (TRSJTUSM), immediately placed in liquid nitrogen, and stored at -80°C. This study was approved by the Ethics Committee of TRSJTUSM (No. 2017-003-01). All patients provided written informed consent and adhered to the Declaration of Helsinki.

### Data collection

RNA-seq data for 33 cancers from TCGA were obtained from the University of California Santa Cruz (UCSC, https://xena.ucsc.edu/) xena, and clinical data for the TCGA-COAD dataset were obtained. Somatic mutation data in COAD patients were obtained from The Cancer Genome Atlas (TCGA, https://portal.gdc.cancer.gov/) database. The RNA-seq data and clinical data of GSE39582, GSE161158 and GSE78220 were obtained from the Gene Expression Omnibus (GEO, https://www.ncbi.nlm.nih.gov/geo/) database. Consensus molecular subtypes (CMS) of COAD patients in GSE39582 were obtained from data from previously published studies (13). RNA-seq data and matched clinical data from patients in the IMvigor210 cohort of advanced urothelial carcinoma treated with atezolizumab from previously published studies (14). All RNA-seq data were investigated in log _2_ (FPKM+1). Patients with an overall survival of less than 30 days were excluded from the prognostic correlation analysis.

### Identification of key genes related to angiogenesis

The 36 ARG were obtained from h.all.v7.5.1.symbols.gmt (http://www.gsea-msigdb.org/gsea/msigdb/cards/HALLMARK_ANGIOGENESIS). Based on RNA-seq data of TCGA-COAD samples (471 tumors *vs*. 41 normal samples) obtained from UCSC xena, 35 ARG were identified using the limma package (PRG2 expression values with an average value of less than 0.2 were excluded) of differentially expressed genes (P < 0.05, |log _2_ FC| > 1). Key genes were then identified by a univariate Cox regression model.

### ARGPI construction and verification

The ARGPI was constructed based on the results of the univariate Cox regression model. Briefly, the ARGPI for each sample is calculated by multiplying the expression values of key genes by their weight coefficient (coef values) in the Cox model and adding them together. Its ability to predict patient prognosis in the TCGA-COAD, GSE39582, GSE161158 and the TRSJTUSM Cohort. In addition, the prognostic value of ARGPI in 32 cancers was validated using the Kaplan-Meier survival curve analysis.

### RNA extraction and qRT-PCR analysis

Related experiments were performed as previously described (15). Briefly, total RNA was extracted from frozen tissue using RNAiso Plus reagent (Takara Bio, Japan), and RNA was reverse transcribed to cDNA using PrimeScript™ RT Master Mix (Takara Bio, Japan). Quantification was performed by SYBR-Green qPCR Master Mix (Vazyme Bio, China) and normalized to β-actin. Relative quantification of PCR experimental data was performed using the 2−ΔΔCt method. PCR reaction program: preheat at 95°C for 30s, and then enter the amplification cycle. Denaturation at 95°C for 30s, renaturation at 55°C for 30s, and then maintained at 72°C for 60s to complete one cycle. The number of cycles was set to 40 times. Finally, the program was terminated after 5 minutes at 72°C.

The β-actin primer sequences are as follows, forward: CCCTGGAGAAGAGCTACGAG, reverse: GGAAGGAAGGCTGGAAGAGT.

The VEGFA primer sequences are as follows, forward: AGGGCAGAATCATCACGAAGT, reverse: AGGTCTCGATTGGATGGCA.

The JAG2 primer sequences are as follows, forward: AGCCATGCCTTAACGCTTTT, reverse: CACACACTGGTACCCGTTCA.

The TIMP1 primer sequences are as follows, forward: CGCAGCGAGGAGGTTTCTCAT, reverse: GGCAGTGATGTGCAAATTTCC.

### Clinical significance of ARGPI and construction of nomogram

To determine whether ARGPI is an independent prognostic marker affecting OS in patients with COAD, we performed univariate and multivariate Cox regression analyses, respectively. Based on ARGPI, we combined multiple clinical indicators to construct a nomogram and used it to predict 1-, 3-, and 5-year OS in COAD patients. Calibration curve was used to evaluate the consistency between nomogram predictions and actual values, and ROC curve was used to evaluate the AUC value of the nomogram in predicting OS of COAD patients at 1-, 3-, and 5-year.

### Gene set enrichment analysis

The TCGA-COAD samples were divided into high and low ARGPI groups by median ARGPI value. Based on the gene sets “c2.cp.kegg.v7.4.symbols.gmt” and “c5.go.v7.4.symbols.gmt”, the biological processes (BP) and signaling pathways of different ARGPI groupings affecting COAD were analyzed by GSEA. GSEA is a classic tool for elucidating biological functions across different subgroups of patients through genomic expression data (16).

### Immune cell infiltration analysis

xCell is a novel method to infer 64 immune and stromal cells based on gene signatures combined with gene set enrichment dominance and deconvolution methods (17). We calculated the infiltration levels of 64 immune and stromal cells in TCGA-COAD samples using xCell.

### Mutation spectrum analysis

Somatic mutation data from TCGA-COAD patients were analyzed based on the maftools package (18). We first analyzed the mutational spectrum of angiogenesis-related genes in TCGA-COAD patients. Secondly, according to the median value of ARGPI, TCGA-COAD patients were divided into high ARGPI group and low ARGPI group, and the top 10 most easily mutated genes in different groups were discussed respectively. and calculated the tumor mutational burden (TMB) for each sample, TMB typically represents the number of nonsynonymous mutations per megabase in somatic cells (19).

### Immunotherapy responsiveness and drug sensitivity analysis

We first analyzed the correlation of ARGPI with PDL1 and TMB. And use the TIDE algorithm to predict and predict cancer immunotherapy response (20), and analyze the difference of TIDE-related scores in different APGRI groups. Finally, the ability of ARGPI to predict immunotherapy response was validated using two immunotherapy cohorts (IMvigor210 cohort and the GSE78220 cohort). In this study, Immunotherapy efficacy indicators include complete remission (CR), partial remission (PR), stable disease (SD) and disease progression (PD). CR and PR were classified as immunotherapy response, SD and PD as immunotherapy nonresponse.

The IC50 values of 88 common chemotherapy and targeted drugs were analyzed using the pRRophetic package to study the sensitivity of different APGPI groups to different drugs.

### Statistical analysis

The TMB is obtained by processing the PERL programming language (v5.32.1). All data were analyzed in R language (R version 4.1.0). The Spearman method was used for correlation analysis, and the Wilcoxon test was used to analyze the differences between different ARGPI groups. Comparison of the distribution of CMS in different ARGPI groups by chi-square test. P<0.05 represents statistical significance.

## Result

### Construction and verification of ARGPI in COAD

**Figure 1:**
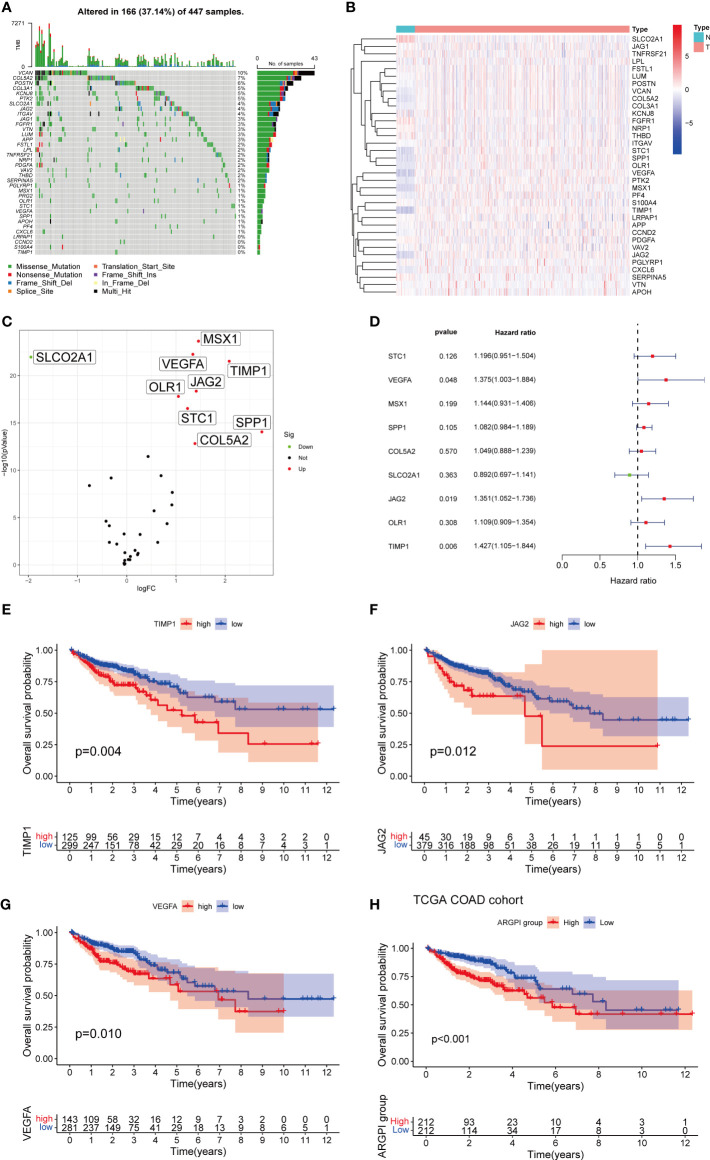
Construction of ARGPI. **(A)** 35 ARG mutation spectrum. **(B, C)** Heatmap and volcano plot of expression differences of 35 ARG between COAD and normal tissues. **(D)** Univariate Cox Analysis of 10 DEARG. **(E–G)** Kaplan-Meier survival analysis of three significant ARG in univariate Cox analysis. **(H)** Kaplan-Meier survival analysis of ARGPI subgroups in the TCGA-COAD cohort.

First, we determined the somatic mutation of 35 ARG in COAD. As shown in **Figure 1A**, 167 of the 433 COAD samples had somatic mutations, of which VCAN was the ARG with the highest somatic mutation frequency (10%), followed by COLA5A and POSTN. Second, we obtained 471 COAD tissues and 41 normal tissues from TCGA, and identified 9 differentially expressed ARG (DEARG), including 8 up-regulated DEARG and 1 down-regulated DEARG. The associated heatmaps and volcano maps are shown in **Figures 1B, C**.

To identify prognostic genes affecting COAD survival, we performed a univariate Cox regression analysis of OS for 9 DEARGs. The results showed that only three genes (VEGFA, JAG2, and TIMP1) significantly affected OS in COAD patients (**Figures 1D–G**). We then constructed a prognostic index for all cancer samples based on the results of univariate Cox regression analysis (The detailed results of univariate Cox regression analysis are listed in **Supplementary Table S1**). ARGPI formula =coef of VEGFA*expression level of VEGFA+ coef of JAG2 * expression level of JAG2 + coef of TIMP1 * expression level of TIMP1. The COAD patients were divided into high ARGPI group and low ARGPI group by the median ARGPI value, and the results showed that the OS of the high ARGPI group was significantly lower than that of the low ARGPI group (**Figure 1H**).

### The external cohort validation of ARGPI

**Figure 2:**
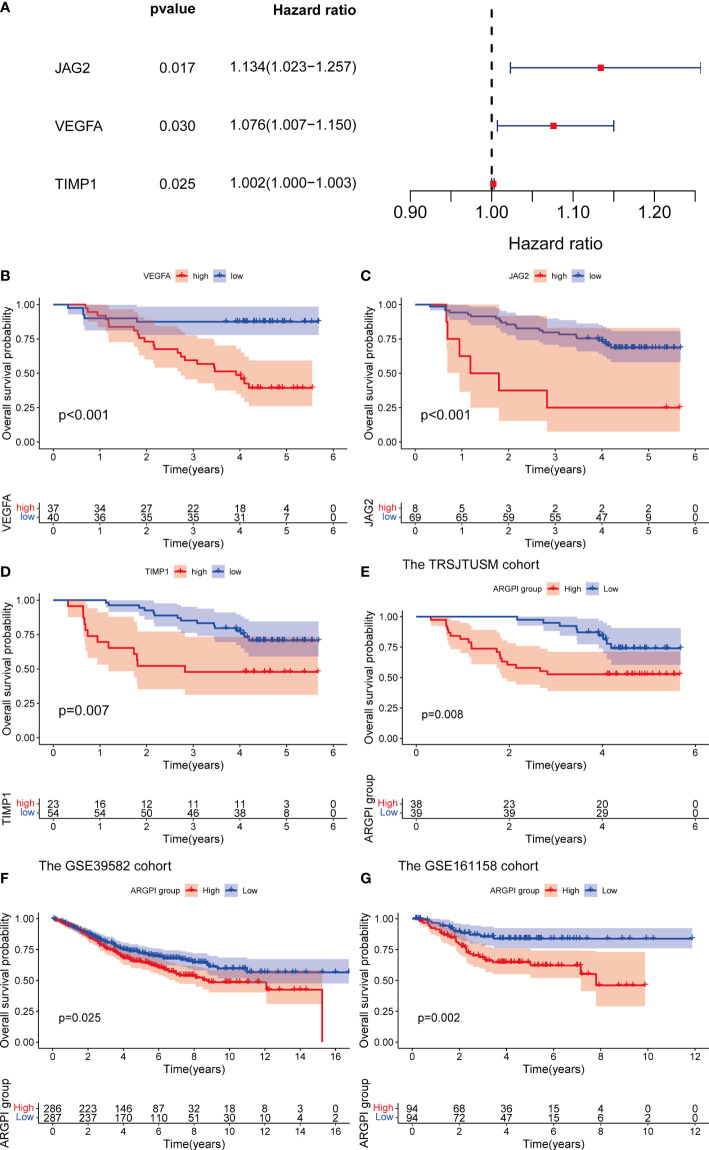
Verification of ARGPI. **(A)** Univariate Cox Analysis of JAG2, VEGFA and TIMP1 in TRSJTUSM Cohort. **(B–D)** Kaplan-Meier survival analysis of JAG2, VEGFA and TIMP1 in TRSJTUSM Cohort. **(E)** Kaplan-Meier survival analysis of ARGPI subgroups in the TRSJTUSM cohort. **(F)** Kaplan-Meier survival analysis of ARGPI subgroups in the GSE39582 cohort. **(G)** Kaplan-Meier survival analysis of ARGPI subgroups in the GSE161158 cohort.

In order to verify the stability and universality of ARGPI to guide prognosis. We first examined the expression of key ARGs using samples from the TRSJTUSM cohort. Univariate Cox regression model and Kaplan-Meier survival curve analysis showed that all three key ARGs significantly affected OS in CRC patients (**Figures 2A–D**). The ARGPI of the TRSJTUSM cohort was then constructed according to the above ARGPI formula. Similarly, CRC patients were divided into high-ARGPI group and low-ARGPI group according to the median ARGPI, and the results showed that the OS of the low-ARGPI group was significantly better than that of the high-ARGPI group (**Figure 2E**), which is consistent with the results of the TCGA-COAD dataset of. More importantly, consistent results were shown in GSE39582 and GSE161158, with significantly lower OS in the high ARGPI group than in the low ARGPI group (**Figures 2F, G**). Finally, we validated the prognostic value of ARGPI in 32 cancer types, and the results showed that ARGPI exhibits potential prognostic value in 22 cancer types (**Supplementary Figure 1**).

### Construction of a nomogram to predict the prognosis of patients with COAD

**Figure 3:**
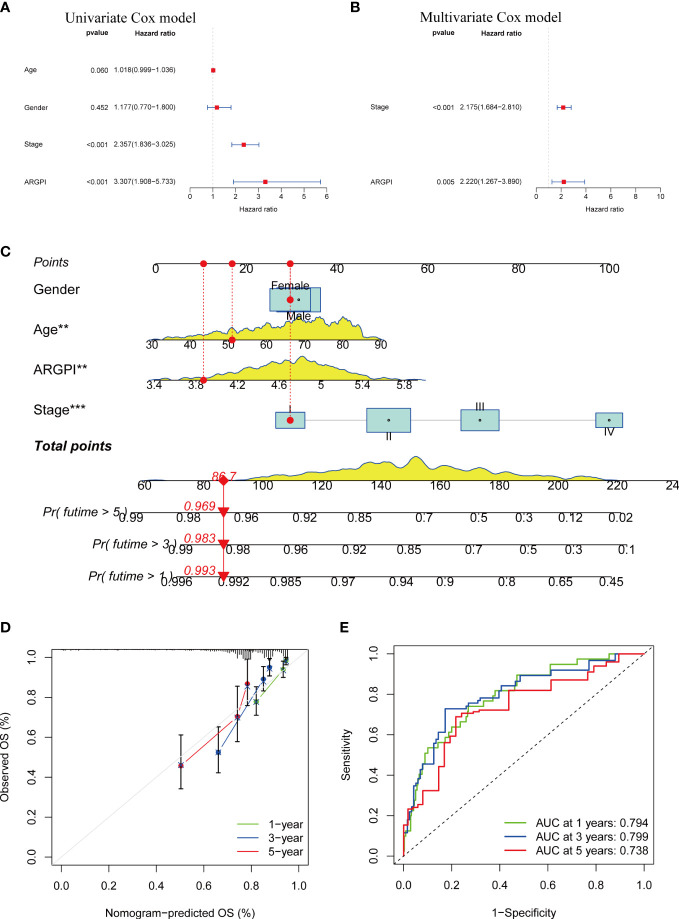
Construction of a nomogram. **(A, B)** Univariate and multivariate Cox analysis of clinicopathological factors and ARGPI in TCGA-COAD Cohort. **(C)** A nomogram based on ARGPI and clinicopathological factors to indicate OS in COAD patients. **(D, E)** Calibration curves and ROC curves were used to assess the robustness and accuracy of nomograms in predicting 1-, 3-, and 5-year OS in COAD patients. ** p < 0.01, and *** p < 0.001.

First, we fitted into the clinicopathological features of COAD patients. Univariate Cox regression analysis showed that tumor stage and ARGPI were significantly associated with prognosis in COAD (**Figure 3A**). Multivariate Cox regression analysis confirmed that tumor stage and ARGPI were independent prognostic factors affecting COAD patients after adjusting for other clinicopathological factors (**Figure 3B**). Furthermore, we validated this conclusion in the TRSJTUSM cohort. Results show that tumor stage and ARGPI are independent prognostic factors for colorectal cancer patients (**Supplementary Figures 2A, B**). Due to the high correlation between ARGPI and patient prognosis, we constructed a nomogram combining clinical parameters. The nomograms we constructed were designed to assess 1-, 3-, and 5-year OS in COAD patients (**Figure 3C**). Calibration curves revealed a high consistency between the predicted values and actual values of nomograms for 1-, 3-, and 5-year OS in COAD patients (**Figure 3D**). In addition, we analyzed the AUC values of the nomogram for predicting OS at 1-, 3-, and 5-years by ROC curves. As shown in **Figure 3E**, the nomogram showed excellent predictive power for predicting the prognosis of COAD patients at 1, 3, and 5 years (AUC values of 0.794, 0.799, and 0.738, respectively).

### GSEA

**Figure 4:**
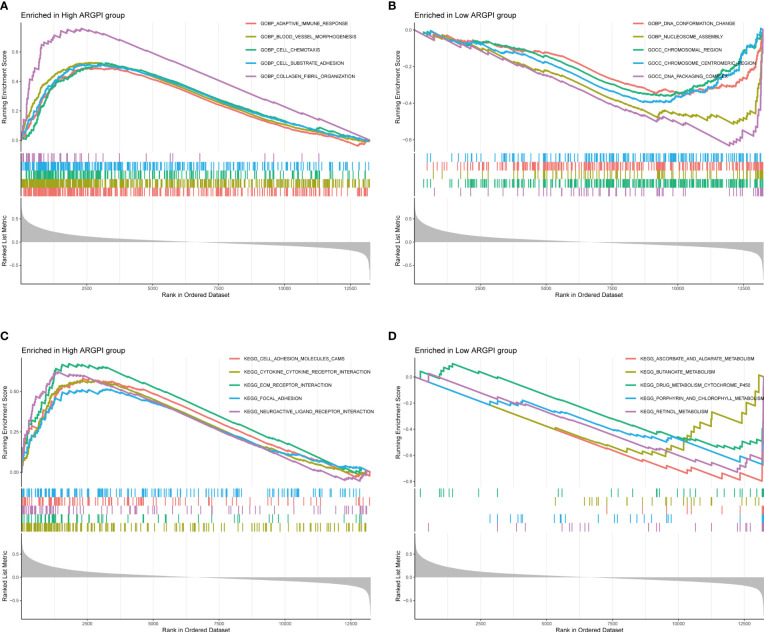
Gene Set Enrichment Analysis of ARGPI. **(A, B)** The 5 biological processes that were most significantly enriched in the high ARGPI group and the low ARGPI group, respectively. **(C, D)** The 5 signal pathways that were most significantly enriched in the high ARGPI group and the low ARGPI group, respectively.

To gain insight into the biological processes and signal transduction pathways that ARGPI may affect in COAD, we divided patients into high and low ARGPI groups with median ARGPI values and performed GSEA. First, we explored the biological process by which ARGPI might affect COAD, and the results showed that the BPs involved in the high ARGPI group were mainly enriched in GOBP ADAPTIVE IMMUNE RESPONSE, GOBP BLOOD VESSEL MORPHOGENESIS, GOBP CELL CHEMOTAXIS, GOBP CELL SUBSTRATE ADHESION, GOBP COLLAGEN FIBRIL ORGANIZATION (**Figure 4A**). The BPs involved in the low ARGPI group were mainly enriched in GOBP DNA CONFORMATION CHANGE, GOBP NUCLEOSOME ASSEMBLY, GOCC CHROMOSOMAL REGION, GOCC CHROMOSOME CENTROMERIC REGION, GOCC DNA PACKAGING COMPLEX (**Figure 4B**). Second, we explored the signaling pathways by which ARGPI may affect COAD. The results showed that the signaling pathways affected by the high ARGPI group were mainly enriched in KEGG CELL ADHESION MOLECULES CAMS, KEGG CYTOKINE CYTOKINE RECEPTOR INTERACTION, KEGG ECM RECEPTOR INTERACTION, KEGG FOCAL ADHESION, KEGG NEUROACTIVE LIGAND RECEPTOR INTERACTION (**Figure 4C**). The signaling pathways affected by the low ARGPI group were mainly enriched in KEGG ASCORBATE AND ALDARATE METABOLISM, KEGG BUTANOATE METABOLISM, KEGG DRUG METABOLISM CYTOCHROME P450, KEGG PORPHYRIN AND CHLOROPHYLL METABOLISM, KEGG RETINOL METABOLISM (**Figure 4D**). Detailed results of GSEA are presented in **Supplementary Table S2**.

### Correlation between ARGPI and immune cell infiltration

**Figure 5:**
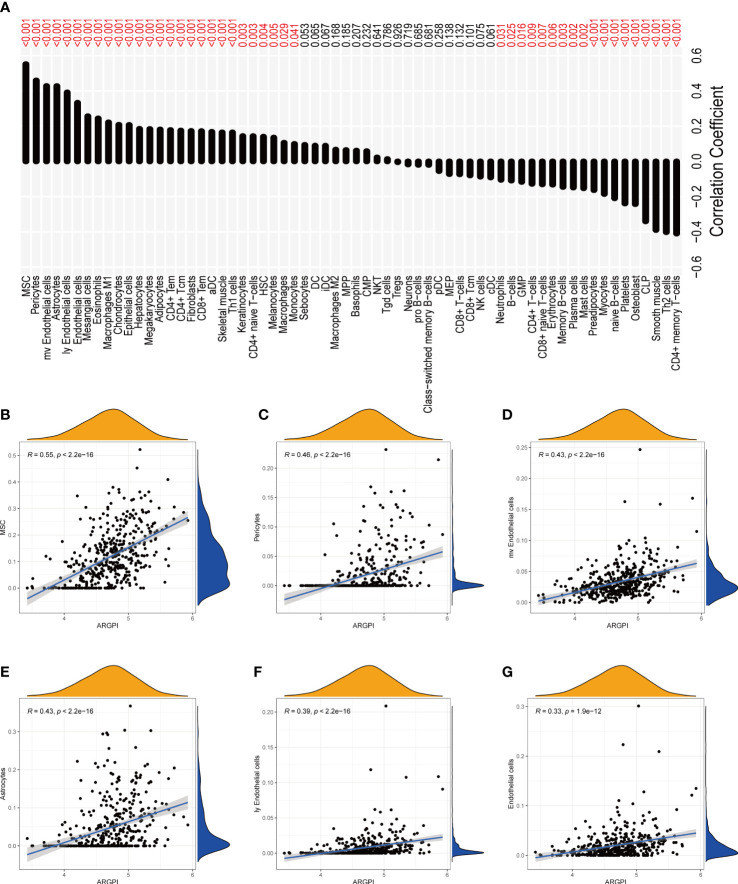
Correlation between ARGPI and immune cell infiltration. **(A)** Correlation histogram of ARGPI with 64 kinds of immune cells and stromal cells. **(B–G)** Scatter plot of 6 immune cell correlations most significantly associated with ARGPI.

To determine whether ARGPI affects the level of immune cell infiltration in COAD samples, we assessed the infiltration levels of 64 immune-related cells in COAD samples using xCell. Using Spearman to analyze the correlation of ARGPI with immune cell infiltration, the results showed that ARGPI was associated with 45 out of 64 immune cell types (p < 0.05), including a positive correlation with 27 immune cell infiltration and 18 immune cell infiltration was negatively correlated (**Figure 5A**). Among them, Mesenchymal stem cell (MSC) were the immune cells with the strongest correlation with ARGPI (**Figure 5B**), followed by Pericytes (**Figure 5C**), mv Endothelial cells (**Figure 5D**), Astrocytes (**Figure 5E**), ly Endothelial cells (**Figure 5F**), Endothelial cells (**Figure 5G**).

### Correlation between ARGPI and CMS

**Figure 6:**
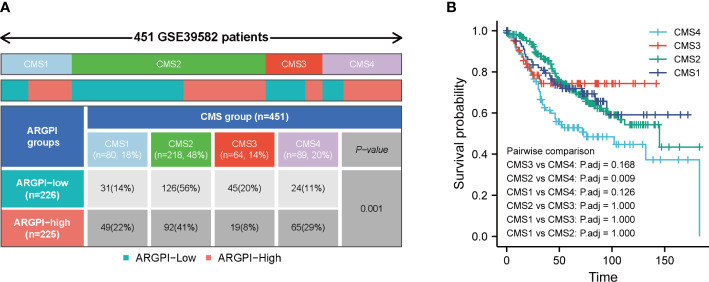
Distribution of consensus molecular subtypes (CMS) in different ARGPI subgroups **(A)** and prognostic analysis of CMS **(B)**.

Previous studies have shown that COAD patients can be classified into 4 CMS. In short, CMS1 is known as the “MSI-like” subtype. CMS2 is known as the “Canonical” subtype. CMS3 is known as the “Metabolic” subtype. CMS4 is known as the “mesenchymal” subtype (13). We obtained the classification of consensus molecular subgroups of GSE39582 from previous studies, and further analyzed the distribution between ARGPI groupings and CMS, as shown in **Figure 6A**, there were significantly more CMS1 and CMS4 in the high ARGPI group than in the low ARGPI group, especially CMS4. While the low ARGPI group had significantly more CMS2 and CMS3 than the high ARGPI group. In addition, we also analyzed the prognosis in different CMS groups, and the results showed that the prognosis of the CMS4 phenotype was significantly worse, especially relative to the CMS2 phenotype (**Figure 6B**).

### Association of ARGPI with PDL1, TMB, MSI and TIDE scores

**Figure 7:**
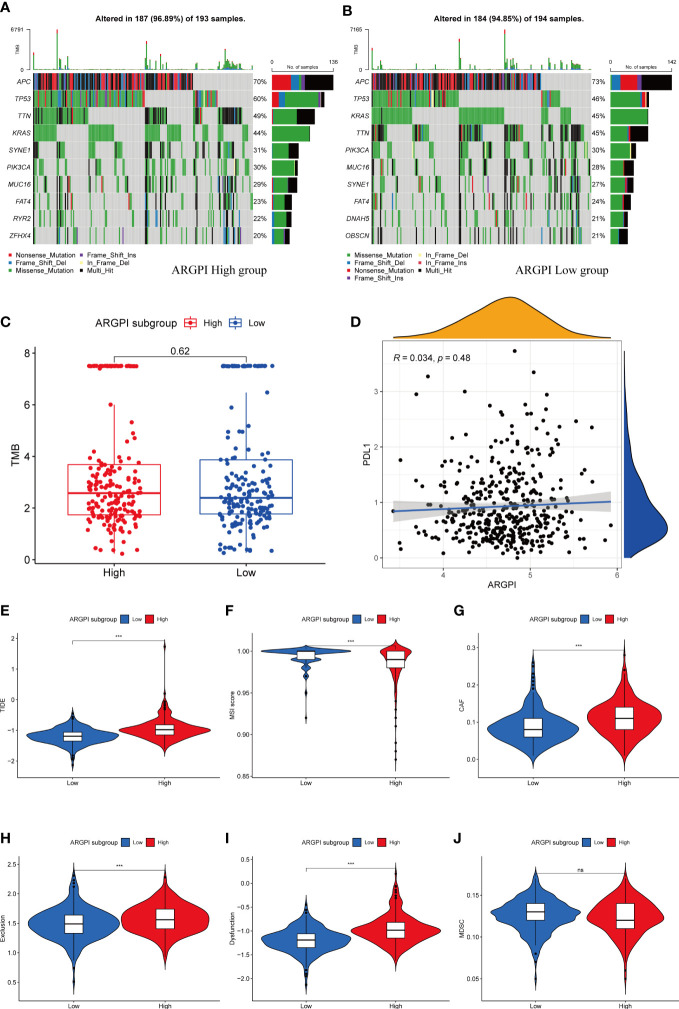
Association of ARGPI with markers for predicting ICI response. **(A, B)** The 10 most mutated genes in TCGA-COAD samples in the high and low ARGPI groups. **(C)** Differences in TMB in different ARGPI groups. **(D)** Correlation between ARGPI and PDL1 expression. **(E–J)** TIDE, MSI score, CAF, T-cell Exclusion and dysfunction scores, and MDSC in different ARGPI groups. “ns” represents not significant and “***” represents p < 0.001.

Numerous studies have shown that PDL1, TMB and Microsatellite instability (MSI) are important predictors of immunotherapy response (21). First, we explored the gene mutation between different ARGPI groups, as shown in **Figures 7A, B**, we showed the 10 most easily mutated genes in different ARGPI groups. The mutation rates of APC, TP53, TTN and KRAS were higher than 40% in both groups. TP53, RYR2 and ZFHX4 were more common in the high ARGPI group. DNAH5 and OBSCN were more common in the low ARGPI group. We next discussed the association between ARGPI and PDL1 and TMB, and the results showed that ARGPI was not significantly correlated with neither PDL1 nor TMB (**Figures 7C, D**). Finally, we evaluated the potential clinical benefit of immunotherapy in different ARGPI groups by using TIDE. A lower TIDE score represents a lower possibility of immune evasion. That is, the lower the TIDE score, the greater the benefit of the patient from immune checkpoint inhibitor (ICI) treatment. In our results, the TIDE score in the low ARGPI group was significantly lower than that in the high ARGPI group (**Figure 7E**), implying that patients in the low ARGPI group had a higher clinical benefit from ICI than those in the high ARGPI group. In addition, compared with the high ARGPI group, patients in the low ARGPI group had higher MSI scores (**Figure 7F**) and lower cancer-associated fibroblasts (CAF), Exclusion, and Dysfunction (**Figures 7G–I**). However, there was no significant difference in Myeloid-derived suppressor cell (MDSC) between the two groups (**Figure 7J**).

### Benefits of ICI therapy in different ARGPI subgroups

**Figure 8:**
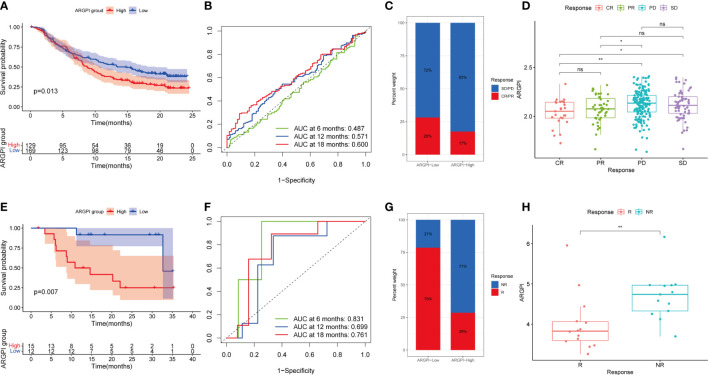
Validation of ARGPI ability to predict therapeutic benefit in ICI therapy. **(A)** Kaplan-Meier survival analysis of ARGPI subgroups in the IMvigor210 cohort. **(B)** ROC analysis of OS with ARGPI at 6-, 12- and 18-months follow-up in the IMvigor210 cohort. **(C)** Clinical response rates to ICI immunotherapy in the high and low ARGPI groups in the IMvigor210 cohort. **(D)** ARGPI distribution in different ICI immunotherapy clinical response status groups in the IMvigor210 cohort. **(E)** Kaplan-Meier survival analysis of ARGPI subgroups in the GSE78220 cohort. **(F)** ROC analysis of OS with ARGPI at 6-, 12- and 18-months follow-up in the GSE78220 cohort. **(G)** Clinical response rates to ICI immunotherapy in the high and low ARGPI groups in the GSE78220 cohort. **(H)** ARGPI distribution in different ICI immunotherapy clinical response status groups in the GSE78220 cohort. “ns” represents not significant, “*” represents p < 0.05 and “**” represents p < 0.01.

Inhibition of immune checkpoints using anti-PD1 and anti-CTLA4 monoclonal antibodies has become a mainstay option in anticancer therapy with unprecedented (22–24). Next, we explored the prognostic value of ARGPI for ICI therapy by combining the IMvigor210 and GSE78220 cohorts to assess clinical benefit. In the IMvigor210 cohort, the prognosis was worse in the high ARGPI group (**Figure 8A**), which is consistent with the results of the COAD cohort. The ROC curve of ARGPI in predicting OS of patients in IMvigor210 showed certain accuracy, with AUCs of 0.487, 0.571 and 0.600 for predicting OS at 6 months, 12 months and 18 months, respectively (**Figure 8B**). In addition, significantly more patients responded to ICI treatment in the low ARGPI group (**Figure 8C**). Likewise, the ARGPI was significantly lower in the ICI response group than in the ICI nonresponse group (**Figure 8D**). The results obtained based on IMvigor210 were further confirmed in GSE78220. First, patients in the high ARGPI group had a worse prognosis (**Figure 8E**), and the ROC curve for predicting patient OS in GSE78220 showed higher accuracy, with AUCs of 0.831, 0.699, and 0.761 for predicting OS at 6, 12, and 18 months, respectively (**Figure 8F**). Likewise, fewer patients responded to ICI treatment in the high ARGPI group (**Figure 8G**), and ARGPI was lower in the ICI response group (**Figure 8H**). Taken together, our data strongly suggests that ARGPI has a strong ability to assess response to ICI therapy.

### Drug sensitivity analysis

**Figure 9:**
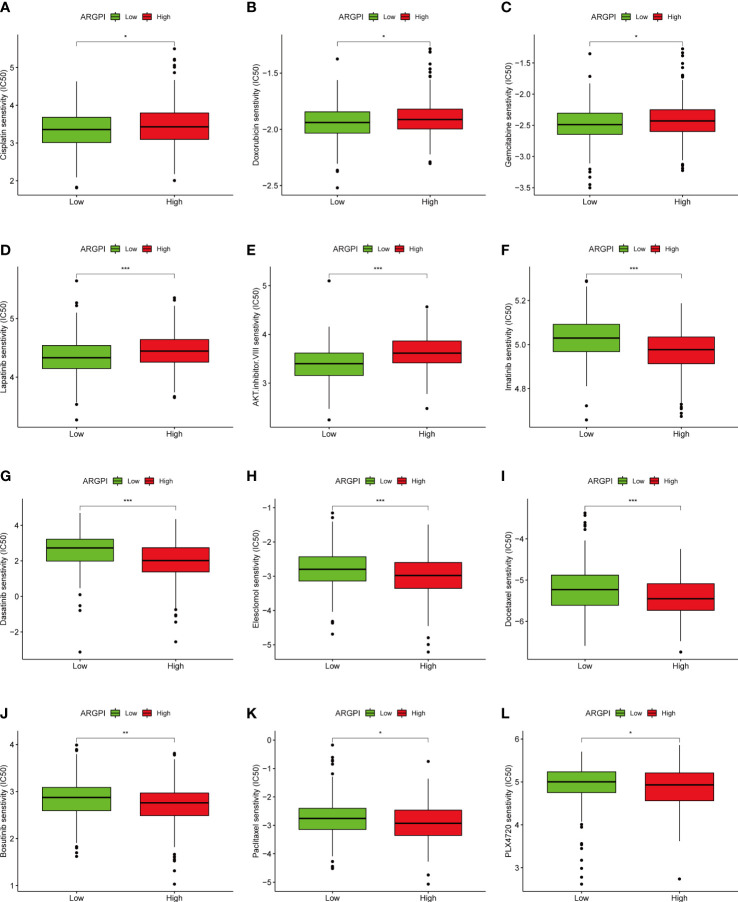
The relationship between ARGPI and treatment sensitivity. **(A–L)** Differences in sensitivity of 12 common drugs among different ARGPI groups. “*” represents p < 0.05, “**” represents p < 0.01, and “***” represents p < 0.001.

Chemotherapy and targeted therapy are also important treatment strategies for patients with metastatic CRC (25, 26). Therefore, to understand whether ARGPI could serve as an ability to predict sensitivity to chemotherapy and targeted therapy in COAD patients, we assessed IC50 values for 88 common drugs in TCGA-COAD patients using the pRRophetic package. We found that COAD patients with different ARGPI groups had significantly different IC50s for 51 drugs (**Supplementary Table 3**). We show differences in the sensitivity of 20 common chemotherapy/targeted agents across different ARGPI groups. The low ARGPI group may be more sensitive to drugs such as Cisplatin, Doxorubicin, Gemcitabine, Lapatinib and AKT.inhibitor.VIII (**Figures 9A–E**). The high ARGPI group may be more sensitive to drugs such as Imatinib, Dasatinib, Elesclomol, Docetaxel, Bosutinib, Paclitaxel, and PLX4720 (**Figures 9F–L**). Overall, ARGPIs were strongly associated with drug sensitivity.

## Discussion

Current studies have fully demonstrated the critical role of angiogenesis in promoting tumor growth and metastasis (27, 28). Pro-angiogenic-related factors not only promote pathological angiogenesis in cancer, but also lead to immunosuppression (27). Conversely, innate and adaptive immune cells regulate angiogenesis in the tumor microenvironment (TME) by producing and releasing numerous pro-angiogenic factors (27, 29). Unfortunately, the clinical benefit of antiangiogenic agents alone in CRC patients is limited (30). Given that angiogenesis and immune microenvironment are closely related and cross-talk, combination therapy based on classical anti-angiogenic therapy and ICI therapy, which has achieved major breakthroughs in the field of cancer treatment, has received extensive clinical attention (31–34). At present, a small number of studies have begun to focus on the clinical application of anti-angiogenic agents and ICIs in CRC (35). However, effective predictive markers based on angiogenesis-related factors for treatment selection in patients with COAD have not yet been developed. Therefore, the necessity and urgency of exploring ARG as prognostic markers and therapeutic targets in COAD patients is more prominent. In this study, we analyzed the DEARG on the basis of the TCGA-COAD cohort, we obtained 9 DEARG, and further univariate Cox regression model determined that three genes were influencing prognostic factors for OS in COAD patients, and ARGPI was constructed based on these three genes. In the TCGA-COAD, GSE39582, GSE161158 and the TRSJTUSM cohorts, survival was lower in the high ARGPI group and higher in the low ARGPI group. Finally, we validated the prognostic value of ARGPI in 22 cancers by Kaplan-Meier survival curves, although it showed consistent prognostic value with COAD in 15 cancer types, which may suggest that ARGPI may play different roles in different tumors. In conclusion, ARGPI is a good prognostic marker for predicting COAD OS.

ARGPI consists of three genes, VEGFA, JAG2 and TIMP1. VEGFA is one of the main factors driving tumor vascular bed expansion and is significantly upregulated in hypoxia (36). In addition, VEGFA is also associated with antitumor immune responses. VEGFA induces T cell depletion in anti-PD-1-resistant microsatellite stable CRC patients (37). Meanwhile, the first approved angiogenesis inhibitor, bevacizumab, a VEGFA-targeting monoclonal antibody, was initially approved in combination with chemotherapy for the treatment of metastatic CRC (38). Bevacizumab combined with ICI drugs has shown promising preliminary clinical benefits in RCC and NSCLC and is further suitable for patients with CRC with stable microsatellites (39). JAG2 is a transmembrane ligand of the Notch receptor and up-regulated activates the cancer-related Notch signaling pathway (40, 41). Notch signaling can promote and cancer stem cell (CSC) maintenance through crosstalk with VEGF receptors to regulate angiogenesis and influence the TME (41, 42). It has been shown that JAG2 acts as a prognostic factor for colon cancer (43) and is involved in regulating colon cancer cell migration and invasion (44). TIMP1 is a member of the TIMP family with multiple functions (45), and its role in cancer has been controversial (46). Studies have shown that TIMP1 can inhibit apoptosis and promote colon cancer occurrence and metastasis through FAK-PI3K/AKT and MAPK pathways (47). In the calculation formula of ARGPI, the coefficients of VEGFA, JAG2 and TIMP1 are all positive numbers, and ARGPI is positively correlated with VEGFA, JAG2 and TIMP1. In conclusion, ARGPI is closely related to angiogenesis and the immune microenvironment.

After controlling for clinical confounding parameters, ARGPI was shown to be an independent risk factor for OS in COAD patients. Based on ARGPI, combined with clinicopathological parameters, we established a nomogram to predict the prognosis of COAD patients. We validated its accuracy and robustness for 1-, 3-, and 5-year OS prediction with calibration curves and ROC curves. In addition, it is particularly important that we provide an excellent biomarker for predicting OS in COAD patients.

In order to understand the BP and signal transduction pathways in different ARGPI subgroups, it was shown by GSEA that the high ARGPI group mainly related to BP related to angiogenesis and adaptive immunity, and mainly affected cancer-related signaling pathways. This strongly suggests that ARGPI closely links angiogenesis and immunology in COAD. Understanding the TME can help us find new biomarkers for COAD treatment. We first estimated the infiltration levels of 64 immune and stromal cells using the Xcell algorithm. Another important advantage of xCell is the efficient classification of immune cells related to innate and adaptive immunity (17). We found that most immune cell infiltration levels were closely related to ARGPI. Remarkably, we found a significant positive correlation between ARGPI and MSC, pericytes and endothelial cells. Current studies suggest that MSC play an important role in promoting neovascularization (48) and can differentiate into CAF after interacting with cancer cells (49). CAF have been shown to imply a poor prognosis in CRC and promote CSC maintenance and chemoresistance (50, 51). Interaction between pericytes and endothelial cells is a central process regulating blood vessel formation (52). Studies have shown that Tie2 expressed by pericytes controls angiogenesis (53). Furthermore, tumor-associated endothelial cell proliferation-induced angiogenesis is a key factor in carcinogenesis, including CRC (54). Our results imply that ARGPI may affect COAD progression by affecting these immune cells. In terms of COAD immune subtype classification, the high ARGPI group had significantly more CMS4 subtypes than the low ARGPI group, and significantly fewer CMS2 and CMS3 patients. Moreover, the prognosis of patients in the CMS4 subgroup was significantly worse, which may be one of the reasons for the poor prognosis of the AGGPI high expression group. This is consistent with the analysis results obtained from the immune cell correlation analysis.

Next, we explored the relationship between ARGPI and known predictors of immunotherapy response. Such as PDL1, TMB and MSI. In general, PDL1 expression is an effective marker of response to ICI therapy in some tumors, such as non-small cell lung cancer and urothelial carcinoma (55, 56), but is not a good predictor of ICI response in CRC (57). MSI status has been shown to be a strong predictor of response to ICI in CRC patients, and MSI-High means better response to ICI therapy (57). Likewise, TMB may also serve as an emerging biomarker for predicting ICI response in CRC patients (58). MSI-High often means higher TMB, but there is no clear correlation between the two (58). In this study, we first investigated the gene mutation spectrum of different ARGPI groups, and we found that the gene with the largest mutation difference between groups was TP53. The TP53 mutation in the high ARGPI group was significantly higher than that in the low ARGPI group. TP53 is one of the most frequently mutated genes in CRC and is associated with poor prognosis (59). This is consistent with our survival results. Furthermore, we found that ARGPI was not significantly associated with neither PDL1 nor TMB. Therefore, we further used the TIDE score to predict the treatment response of COAD patients to ICI. Interestingly, the different ARGPI groups reflected differences in the clinical benefit of ICI therapy. The low ARGPI group may have a better response to ICI treatment (lower TIDE score). In contrast, the low ARGPI group had higher MSI scores. High MSI may be one of the reasons why the low ARGPI group benefited more from ICI therapy. In addition, the high ARGPI group had higher CAF content and higher T cell Exclusion and Dysfunction scores. Therefore, higher ARGPI-induced immunosuppression may be related to excess CAF or to T cell exclusion and dysfunction. More importantly, we confirmed this notion in two immunotherapy cohorts (IMvigor210 cohort and the GSE78220 cohort). We first analyzed the prognostic value of ARGPI in these two treatment cohorts, and as with COAD, the high ARGPI group implied a worse prognosis. And in the low ARGPI group, the patients with ICI response were significantly higher than those in the high ARGPI group. Likewise, ARGPI scores were significantly lower in the ICI response group. Taken together, our results implicate ARGPI as a promising biomarker for COAD predicting ICI response, offering new possibilities for improving the outcome of immunotherapy in CRC.

5-Fluorouracil-based chemotherapy is the first-line chemotherapy drug for CRC, but the problem of drug resistance has become increasingly serious (60). Reuse of old drugs is an important strategy in the development of anti-tumor drugs, which has the advantages of cost saving and drug safety. This study identified potentially sensitive drugs in patients in different ARGPI groups. The combination of these drugs with anti-vascular survival agents and ICI therapy may help to improve the efficacy of treatment and reduce the occurrence of drug resistance.

Although our model is beneficial for assessing patient prognosis and guiding clinical personalized treatment, there are still shortcomings. For example, TCGA-COAD has fewer clinical factors, and more clinical factors should be included in the future to fully explore the clinical value of ARGPI. It is necessary to conduct extensive prospective studies in the future to gain an in-depth understanding of the prognostic value and therapeutic guidance value of ARGPI in COAD.

## Conclusions

In conclusion, for the first time, we have established a risk model of genetic angiogenesis that can accurately predict the prognosis of COAD patients and analyzed the correlation with TME. ARGPI may be a promising biomarker to guide personalized treatment of COAD. Furthermore, ARGPI consists of only three genes, making it easier to obtain and calculate.

## Data availability statement

The original contributions presented in the study are included in the article/**Supplementary Material**. Further inquiries can be directed to the corresponding authors.

## Ethics statement

The studies involving human participants were reviewed and approved by The Ethics Committee of Tongren Hospital, Shanghai Jiao Tong University School of Medicine. The patients/participants provided their written informed consent to participate in this study. Written informed consent was obtained from the individual(s) for the publication of any potentially identifiable images or data included in this article.

## Author contributions

HP, YC, and WS were responsible for the design of the study. WS, YX, and BZ wrote and completed the manuscript. BZ, MZ, and JC were responsible for data download and analysis. All authors contributed to the article and agreed to the submitted version.

## Funding

This study was funded by the Shanghai Natural Science Foundation (No. 21ZR1458600) and the Shanghai Jiaotong University Medical-Engineering Cross Research Fund (No. YG2022ZD031).

## Conflict of interest

The authors declare that the research was conducted in the absence of any commercial or financial relationships that could be construed as a potential conflict of interest.

## Publisher’s note

All claims expressed in this article are solely those of the authors and do not necessarily represent those of their affiliated organizations, or those of the publisher, the editors and the reviewers. Any product that may be evaluated in this article, or claim that may be made by its manufacturer, is not guaranteed or endorsed by the publisher.

## Supplementary material

Supplementary Figure 1 Kaplan-Meier survival analysis of ARGPI in 32 cancer types.

Supplementary Figure 2
**(A)** Univariate Cox analysis of clinicopathological factors and ARGPI in TRSJTUSM Cohort. **(B)** Multivariate Cox analysis of clinicopathological factors and ARGPI in TRSJTUSM Cohort.
