# Supplementary figures and images for: The prognostic value and immunological role of angiogenesis-related patterns in colon adenocarcinoma
Source: Front Oncol. 2022 Nov 11;12:1003440. doi: 10.3389/fonc.2022.1003440 (PMC9691776; doi:10.3389/fonc.2022.1003440)

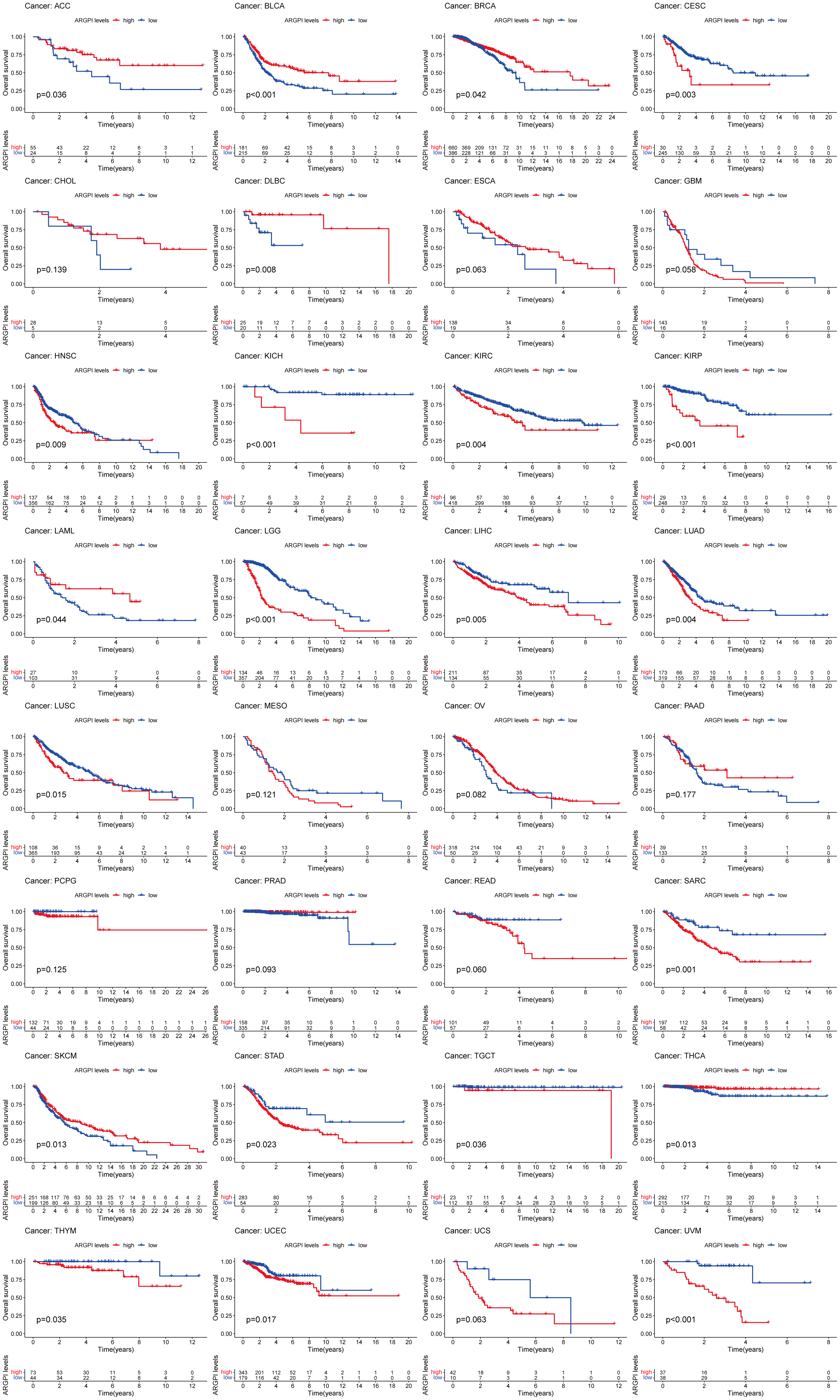

Supplement: Supplementary Figure 1 — Kaplan-Meier survival analysis of ARGPI in 32 cancer types. [file Image_1.tif]

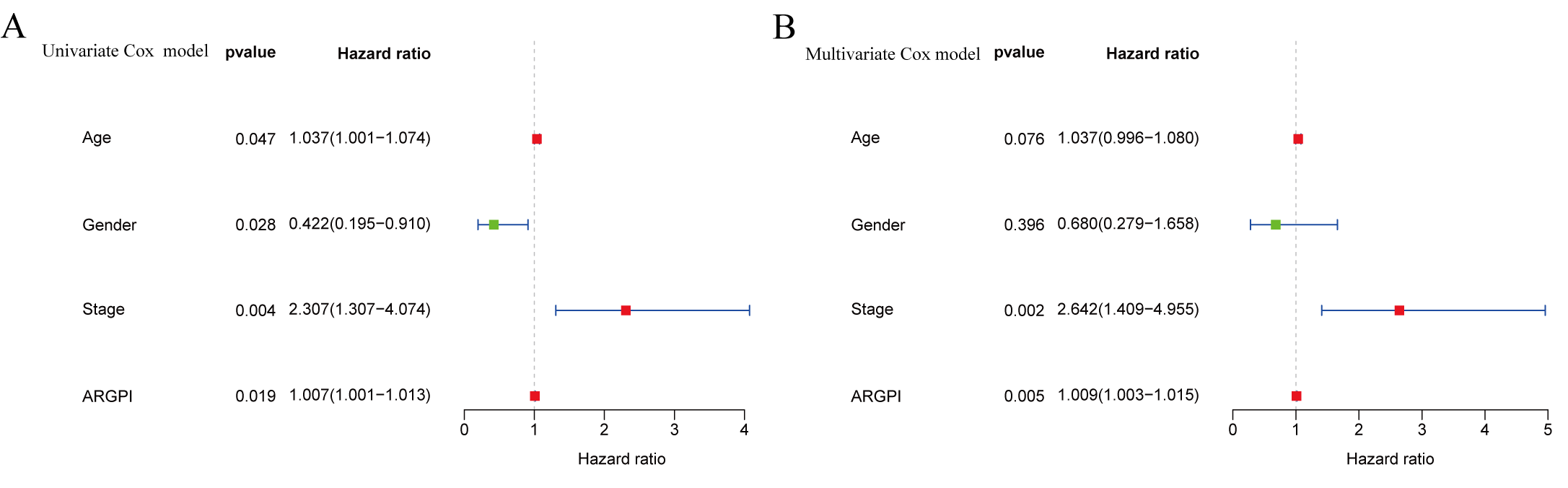

Supplement: Supplementary Figure 2 — (A) Univariate Cox analysis of clinicopathological factors and ARGPI in TRSJTUSM Cohort. (B) Multivariate Cox analysis of clinicopathological factors and ARGPI in TRSJTUSM Cohort. [file Image_2.tif]
